# Supplementary material for: A Proteomic Approach Identified TFEB as a Key Player in the Protective Action of Novel CB2R Bitopic Ligand FD22a against the Deleterious Effects Induced by β-Amyloid in Glial Cells
Source: Cells. 2024 May 19;13(10):875. doi: 10.3390/cells13100875 (PMC11119469; doi:10.3390/cells13100875)
Supplement: Supplementary file 1 [file cells-13-00875-s001.zip › Figure S1.pdf]

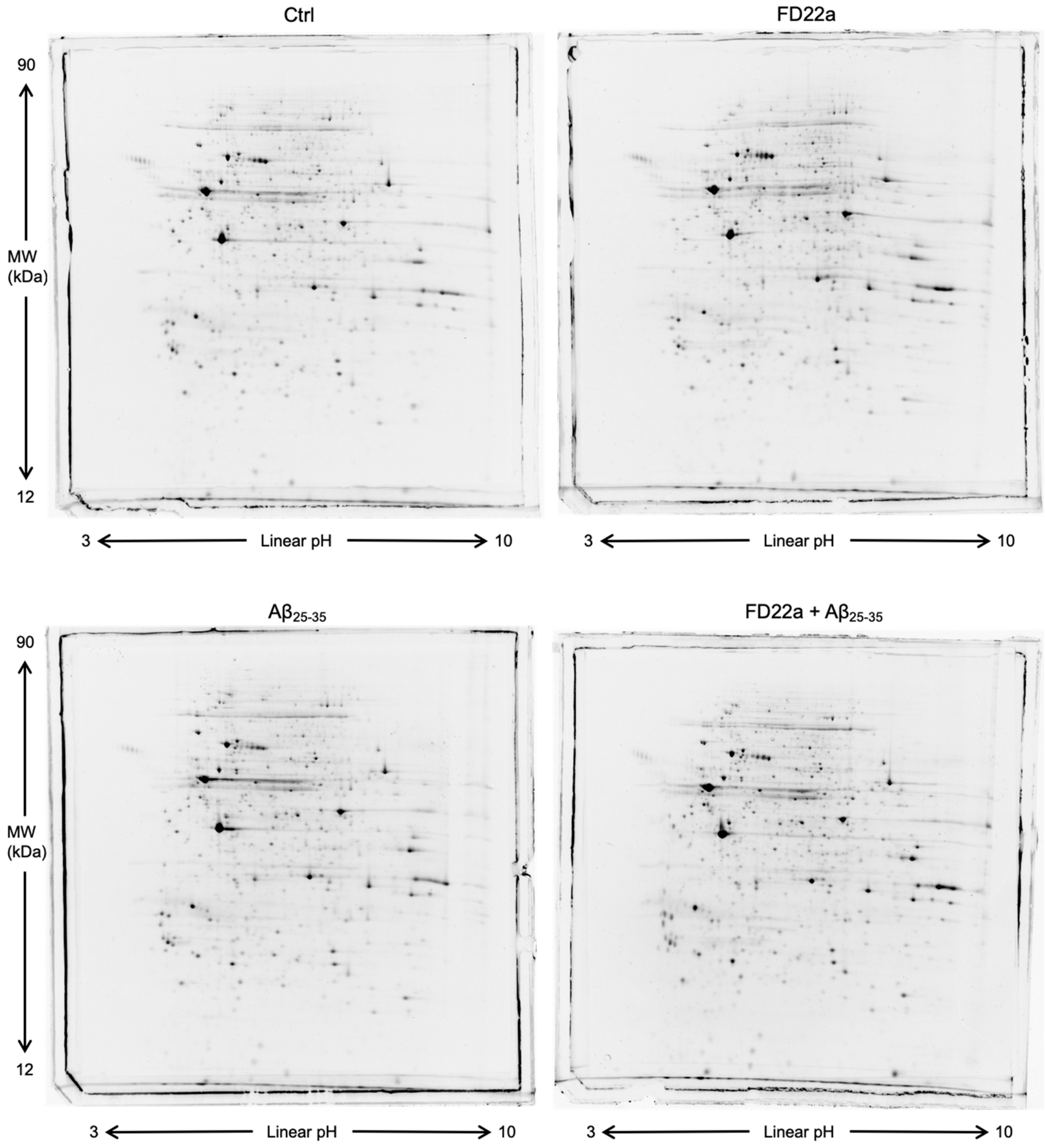

Figure S1. Representative 2DE image of U87-MG proteome in different condition of treatment. Protein extracts were separated in a linear pH 3–10 gradient. SDS-PAGE was performed using 12% acrylamide. Gels were stained with fluorescent dye and acquired by Image Quant L.
